# Estrone-3-Sulphate, a Potential Novel Ligand for Targeting Breast Cancers

**DOI:** 10.1371/journal.pone.0064069

**Published:** 2013-05-22

**Authors:** Nilasha Banerjee, Humphrey Fonge, Andrew Mikhail, Raymond M. Reilly, Reina Bendayan, Christine Allen

**Affiliations:** Department of Pharmaceutical Sciences, Leslie Dan Faculty of Pharmacy, University of Toronto, Toronto, Ontario, Canada; Utrecht University, The Netherlands

## Abstract

The current study investigates the potential of estrone-3-sulphate (E3S) as a ligand for targeting Organic Anion Transporting Polypeptides (OATP), a family of membrane associated uptake transporters, for detection and diagnosis of hormone dependent breast cancers. E3S, an OATP substrate, is a predominant source of tumour estradiol in post-menopausal patients. To assess the potential of E3S as a ligand, distribution of exogenous E3S was determined at the whole body, tumour and cellular levels in murine models of hormone-dependent (MCF-7) and independent (MDA-MB-231) breast cancers. The highest levels of tumour uptake were observed at 6 h post injection (p.i) with significant difference (p = 0.04) between the level in MCF-7 (13.9±3.1%ID/g) and MDA-MB-231 (10.4±1.1%ID/g) (%ID/g: percentage of the total injected dose per gram tissue). The highest tumour-to-blood ratios (MCF-7∶7.4±1.2; MDA-MB-231∶9.1±2.1) were observed at 48 p.i., and highest tumour-to-muscle ratios (MCF-7∶10.7±1.5; MDA-MB-231∶3.8±0.7) were observed at 6 h p.i. Analogous to total tumour uptake, *ex vivo* tumour cell uptake at 2 h p.i. was 6 fold higher in MCF-7 in comparison to MDA-MB-231 tumour cells. Blocking studies, conducted by pre-administration of 100-fold excess E3S, resulted in significantly lower (MCF-7: p = 0.01; MDA-MB-231: p = 0.02) tumour uptake in both xenograft models, suggesting the involvement of an active carrier-mediated process. The expression of OATP1A2 was detected in tumour sections from both xenografts, with significantly higher expression (p = 0.002) in the MCF-7 xenografts. Overall, the higher tumour uptake and tumour-to-muscle ratio, alongside the higher expression of OATP1A2, in the MCF-7 xenograft model suggests the potential of E3S to serve as a novel ligand for targeting hormone dependent breast cancers.

## Introduction

Nearly 75% of estrogen dependent breast cancers are detected in post menopausal women with very low ovarian production of estradiol [1]. Despite the low levels of circulating estrogens, the tumour tissue concentrations of estradiol in these patients have been reported to be significantly higher than that in plasma or in the area of the breast considered normal tissue, suggesting intra-tumoral biosynthesis [2]. Estrone-3-sulphate (E3S), a circulating inactive plasma estrogen, has been reported to serve as the predominant source for tumour estradiol in these post-menopausal patients [3]–[5]. Following its cellular uptake, E3S is desulfated to estrone by estrogen sulphatase which is further converted to estradiol by 17β- hydroxysteroid dehydrogenase [6], [7]. This intracellular production of estradiol then stimulates the proliferation of estrogen dependent tumour cells. As compared to the other unconjugated sources of estrogen (which act as precursors for the aromatase pathway), E3S (precursor for the sulphatase pathway) has about 5 to 10 times higher plasma circulating levels [7]. Moreover, sulphatase activity is 130–200 times higher than aromatase activity [8] and the concentration of sulphatase is three times higher in breast cancer tissues than normal tissues [9]. These observations support that E3S plays a critical role in the proliferation of hormone dependent breast cancers in post-menopausal patients. Given that tumour concentrations of E3S are 2–3 times higher in malignant breast tissues compared to surrounding normal tissues [3] and 2–20 times higher than the plasma circulating levels, [5], [10], [11], E3S may have potential as a ligand for targeting hormone dependent breast cancer in post-menopausal patients.

E3S has a log P value of 1.4 [8], [12], [13] and is unable to easily diffuse freely through the plasma membrane of cells suggesting the role of an active carrier mediated process in cellular uptake of E3S. Indeed, E3S has been recognized as a substrate for the Organic Anion Transporting Polypeptides (OATP), a family of membrane associated uptake transporters belonging to the solute carrier (SLC) superfamily [14], [15]. We have previously demonstrated that some isoforms of the OATP family are involved in the cellular uptake of E3S in various hormone dependent and independent breast cancer cell lines where the OATP mediated E3S transport efficiency was ten fold higher in hormone dependent (MCF-7) in comparison to hormone independent cancer cells [16]. Miki et al. and Meyer zu Schwabedissen et al. reported 10-fold over-expression of one of the OATP isoforms (i.e. OATP1A2) in breast cancer tissues as compared to surrounding normal tissues [17], [18]. Kindla et al. also compared the expression and localization of OATP2B1, OATP3A1 and OATP5A1 in paired samples of normal breast tissue and breast cancer tissue, and reported that while OATP3A1 and OATP5A1 are localized in the plasma membrane of epithelial cells of lactiferous ducts in normal breast tissue, these transporters are highly expressed in the plasma membrane and cytoplasm of breast cancer tissues [19]. The observed high expression and function of certain OATP isoforms indicates that OATPs could be a promising molecular target for breast cancers. As a step towards assessing the potential of E3S as a ligand to target OATP for detection of hormone dependent breast cancers, the current studies examine the biodistribution of exogenous E3S in murine models of breast cancer. To the best of our knowledge, this is the first study to report on the distribution of exogenous E3S at the whole body, tumor and cellular levels, in models of hormone dependent and independent breast cancer.

## Materials and Methods

### Cell Culture and Mouse Xenografts

MCF-7 and MDA-MB-231 cells were purchased from American Tissue Culture Collection (ATCC, Manassas, VA). The cells were cultured at 37°C and 5% CO_2_ in Dulbecco’s modified Eagle medium (DMEM) containing 1% penicillin-streptomycin solution (1000 U/mL, Invitrogen, Grand Island, NY) and supplemented with 10% fetal bovine serum (FBS, Invitrogen, Grand Island, NY). Sub-confluent cells were harvested by trypsinization with trypsin-EDTA (Sigma Aldrich, Mississauga, ON).

Animal experiments were carried out in compliance with the Canadian Council on Animal Care regulations and were approved by the Animal Care Committee of the University of Toronto (AUP # 20008985). Six to eight week-old (20–25 g) female athymic CD-1 ovariectomized nude mice were purchased from Charles River (St. Constant, QC). MDA-MB-231 (5×10^6^) or MCF-7 (1.5×10^7^) cells suspended in saline were injected into the right hind flank of each mouse and allowed to grow into tumour xenografts until they reached a diameter of 5–8 mm. For MCF-7 xenografts, a 17-β-estradiol pellet (0.25 mg/pellet) (Innovative Research of America, Sarasoto, FL) with a 21-day release profile was intradermally implanted into each mouse using a 10 gauge Trocar needle (Innovative Research of America), one day prior to tumour cell inoculation. Following the completion of the 21-day period, an additional 4 days were allowed for clearance of plasma circulating estradiol, prior to initiation of the pharmacokinetics and biodistribution studies in MCF-7 tumour bearing mice. Plasma was collected from both xenograft models one day prior to initiating the pharmacokinetics and biodistribution studies and it was confirmed that the estradiol levels were undetectable in both xenograft models.

Tumor volume was calculated as follows: (length×(width)^∧^2)/2, where length is the longest diameter and width is the shortest diameter perpendicular to length. The mean tumour volumes were 88.46±20.5 and 107.91±25.5 mm^3^ for the MCF-7 and MDA-MB-231 xenografts, respectively.

### Pharmacokinetics and Biodistribution Studies

Non-tumour bearing female athymic CD-1 ovariectomized nude mice (n = 4) were intravenously injected via the tail vein with E3S (3-hydroxyestra-1,3,5(10)-trien-17-one hydrogen sulphate) (Sigma Aldrich, Mississauga, ON) suspended in saline at a dose of 0.25 nmoles E3S/kg (approximately 87.6 ng/kg) body weight spiked with 0.5 µCi/mL [^3^H]-E3S (57.3 Ci/mmol PerkinElmer Life and Analytical Sciences; Waltham, MA). The dose of E3S was determined following consideration of the reported circulating plasma concentrations of E3S (i.e. 1.65 to 3.84 pmol/mL) [6] and the affinity of MCF-7 and MDA-MB-231 cells for E3S (i.e. 6.5±1.9 µM and 46.8±1.7 µM for MCF-7 and MDA-MB-231 cells, respectively, as determined *in vitro* from Km values) [16]. Blood samples were collected via a saphenous vein at different time points (0.08–48 h) using a heparinized capillary tube. Pharmacokinetic parameters including the distribution and elimination half-lives (t_1/2_α and t_1/2_β), volume of distribution at steady-state (V_ss_) and clearance (CL) were estimated by fitting the blood radioactivity vs. time curves to a two-compartment model with i.v. bolus input using Scientist® Ver. 3.0 software (Micromath®, Saint Louis, MO).

Immediately following blood sampling mice were sacrificed under anaesthesia by cardiac puncture and the following tissues and organs were collected and weighed: liver, kidney, spleen and pancreas, bladder, uterus, lung, heart, intestine, stomach, tumour and surrounding muscles. Additionally, two groups of non-tumour bearing mice were injected with 0.25 nmoles/kg E3S (spiked with 0.5 µCi/mL [^3^H]-E3S) and sacrificed at 2 or 6 h post injection (p.i.) for biodistribution studies. Tumour bearing mice (MDA-MB-231 and MCF-7) (n = 5) were also injected with 0.25 nmoles/kg E3S (spiked with 0.5 µCi/mL [^3^H]-E3S) and sacrificed at 2, 6 or 48 h p.i. Excised tumours were divided into two halves, with one half used for biodistribution studies and the other for evaluation of *ex vivo* cellular uptake of E3S. The amount of [^3^H]-E3S in tissues was quantified using a previously described method with minor modifications [20]. Briefly, 1.0 mL of Scintigest Tissue Solubilizer (Thermo Fischer Scientific, Waltham, MA) was added to each test tube containing homogenized tissue samples. The samples were incubated at 55°C for 2 h followed by the addition of 200 µL of 30% (v/v) hydrogen peroxide (Sigma Aldrich, Mississauga, ON). The samples were cooled to room temperature and then transferred into 10 mL scintillation vials containing 3 mL of Ecoscint A (National Diagnostics, Atlanta, Georgia). The samples were counted using a scintillation counter (Beckmam Coulter LS 5000TD, Beckman Instruments Inc., Mississauga, ON). The radioactivity and corresponding total E3S concentrations in samples were expressed as a percentage of the total injected dose per gram tissue (%ID/g).

To determine the specificity of E3S uptake, additional groups of mice (n = 4) bearing MDA-MB-231 or MCF-7 xenografts were injected via the tail vein with 100-fold excess E3S (25 nmoles/kg body weight), two hours prior to the injection of 0.25 nmoles/kg E3S (previously spiked with 0.5 µCi/mL [^3^H]-E3S). The mice were sacrificed 6 h p.i. with blood and tissues collected. Radioactivity and the corresponding E3S concentrations in samples were analyzed as described above.

### *Ex Vivo* Cellular Uptake of ^3^H-E3S in Tumours

One half of the tumour sample was digested to determine the cellular uptake of E3S [21]. Tumours were weighed and thoroughly minced using a surgical scalpel prior to treatment with two portions of disaggregation solution [0.1% collagenase type IV (Sigma Aldrich, Mississauga, ON) and 0.003% DNase I (Sigma Aldrich, Mississauga, ON) in Hank’s buffered salt solution] for 20 min at 37°C with slow agitation. This was followed by centrifugation (4000 rpm) at 4°C for 10 min. The supernatant was discarded and the cell pellets were gently re-suspended in cold PBS containing 0.1% bovine serum albumin type IV (Sigma Aldrich, Mississauga, ON) and 0.2% sodium azide (Sigma Aldrich, Mississauga, ON). The cell pellets were then counted in a scintillation counter. Radioactivity and corresponding total cellular uptake of E3S was expressed as percentage of injected dose (%ID)/g of tumour.

### Analysis of E3S and its Known Metabolites in Plasma

Plasma separated from whole blood collected from tumour bearing mice at 2, 6 or 48 h p.i., was used for HPLC analysis of E3S and its known metabolites. Known metabolites of E3S include estrone and estradiol [22]. Solid phase extraction (SPE) and HPLC analysis of plasma were performed as previously described with minor modifications [22], [23]. SPE was performed using Sep-Pak C_18_ columns (Waters, Mississauga, ON). The column was activated using methanol, followed by water. 100 µL of plasma was then loaded on to the activated column. This was followed by successive washing steps with water and hexane. The analyte was eluted from the column using methanol and then dried under a stream of N_2_. The sample was reconstituted in DMSO:Methanol 1∶1 v/v prior to HPLC analysis. SPE recovery was determined by spiking all samples prior to SPE extraction with 20 µL of each of the standards (0.625 mg/mL E3S, estrone and estradiol). The radioactivity in each eluate was determined by liquid scintillation counting. It was noted that only minor amounts of radioactivity (less than 1%) were present in the hexane and aqueous eluates. The methanol eluates were used for HPLC analysis.

HPLC analysis of E3S and its known metabolites was performed using a Perkin Elmer HPLC system (PerkinElmer, Wellesley, MA) equipped with a UV detector. Separation was performed using a Waters C_18_ reversed-phase HPLC column (XTerra RP-C_18_, 5 µm, 4.6×250 mm I.D.) Analytes were eluted using an isocratic-gradient of 20 mM ammonium sulphate buffer (solvent A) and methanol (solvent B). The mobile phase consisted of solvent A/solvent B; 60/40% for 0–5 min, followed by a linear gradient of 60/40% to 25/75% for 5–45 and 25/75% for 45–50 min at a flow rate of 1.0 mL/min and UV detection at 280 nm. The HPLC eluate from the UV detector was collected every minute for over 50 min in eppendorf tubes and radioactivity in each sample was determined. The radioactivity elution profile for the fractions was plotted and compared with the standard HPLC chromatograms of E3S, estrone and estradiol. The area under the radioactive peaks corresponding to E3S, estrone and estradiol at 2, 6 or 48 h p.i. provided an estimate of the levels of E3S and the known metabolites in plasma at the respective time points.

### Immunohistochemical Analysis

Immunohistochemical staining of tumour sections was performed by the Pathology Research Program (PRP) at the University Health Network (Toronto, ON). Formalin-fixed paraffin-embedded sections (4 µm) of the MCF-7 (n = 6) and MDA-MB-231 (n = 6) xenografts were dewaxed and rehydrated. Antigen retrieval or unmasking was performed by the heat induced epitope retrieval (H.I.E.R) method followed by serum block for 10 min with 10% normal goat serum. Sections were drained and incubated with primary rabbit polyclonal antibodies for anti-OATP1A2 (Sigma Aldrich, Mississauga, ON) and anti-CD31 (Santa Cruz, CA) at 1∶1200 and 1∶1000 dilutions, respectively. These sections were then incubated with Alexa Fluor 488 goat anti-rabbit labelled secondary (Molecular Probes, Burlington, ON) and goat anti-rabbit Cy3 for 60 min for the anti-OATP1A2 and anti-CD31 stained sections, respectively. The slides were then air dried. Expressions of OATP1A2 and CD31 were also examined in human brain tissue sections and in bladder tumour sections, respectively, as positive controls. These sections were then stained with diaminobenzidine (DAB) chromogen and images were acquired using the ScanScope XT (Aperio technologies) at 20X maginification. Slides without any primary antibody staining were also imaged and these served as negative controls. The final images are shown at 10X magnification.

For the OATP1A2 and microvessel densities analysis, microscope images identifying the presence of OATP1A2 and blood vessels (recognized by CD31) were acquired at 10x magnification using an Olympus BX50 upright fluorescence microscope (Olympus, PA) equipped with an EXFO fluorescence illumination source. Images of complete tumour sections were produced by stitching tiled images using MetaMorph software (Molecular Devices Inc., CA). Microvessel and OATP marker densities were measured as the total area of positive signal divided by the surface area of the tumour section analyzed using the “area fraction” tool of the ImageJ software. The unit for immnohistochemical expression was reported as % area [24]. At least six sections collected from independent xenografts were used for determining the mean microvessel and OATP marker densities in each of the tumour models.

### Immunoblot Analysis

Immunoblotting was performed as described previously [16] with minor modifications. Tissues lysates were prepared from three different tumours as previously described. Protein content was determined using an assay kit from Bio-Rad Laboratories (Hercules, CA) and bovine serum albumin (BSA) was the standard. 10 µg of positive control (HEK293/OATP1A2) and 50 µg of the tissue lysates from xenograft tissues (MCF-7 and MDA-MB-231) were loaded. Primary rabbit polyclonal antibody for anti-OATP1A2 (Sigma Aldrich) was used in a 1∶1000 dilution. The blot was also incubated with primary mouse antiactin (AC40) antibody (1∶2000) as a loading control (Santa Cruz). The blots were then incubated for 1.5 h with horseradish peroxidase-conjugated anti-rabbit (1∶15000) or anti-mouse (1∶2000) secondary antibody to detect OATP1A2 and actin, respectively. Signals were enhanced by using a 1∶1 mixture of Reagent A and Reagent B of chemiluminescence SuperSignal West Pico System (Thermo Fisher Scientific, Waltham, MA) and then detected by exposing them to an X-ray film. Densitometric analysis was performed using AlphaDigiDoc RT2 software to quantify relative protein expression.

### Statistical Analyses

All the results were obtained from groups of n≥3 and are presented as mean ± SD. Statistical analysis was performed using Graphpad InStat version 3.0 software (GraphPad Software, Inc., San Diego, CA). Statistical significance was assessed by two-tailed Student's *t* test for unpaired experimental values or one-way analysis of variance (ANOVA) for analysis of repeated measures, as appropriate. *p*<0.05 is considered statistically significant.

## Results

### Pharmacokinetics of [^3^H]-E3S

**Figure 1:**
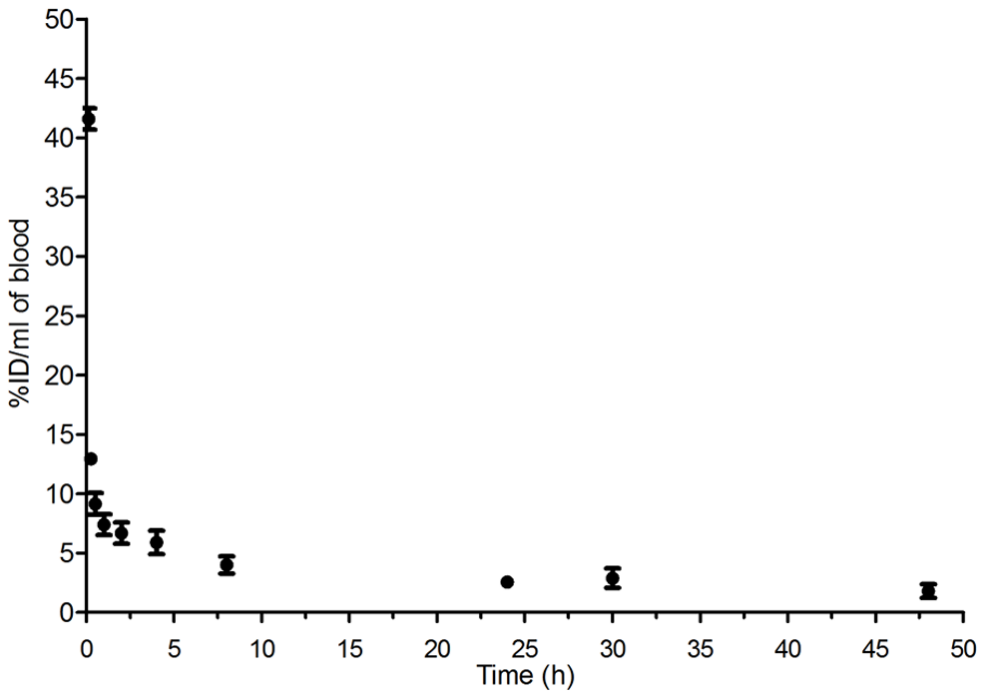
Pharmacokinetic profile of E3S/[^3^H]-E3S administered in healthy mice. Mathematical modeling was performed using the Scientist software (Micromath). Data were fit using a two-compartment model with an i.v. bolus injection, assuming clearance from the central compartment. (%ID/mL, % Injected Dose/mL).

Fig. 1 shows the plasma clearance profile of [^3^H]-E3S in non-tumour bearing athymic CD1 ovariectomized mice. Each mouse was administered 0.25 nmoles/kg E3S spiked with 0.5 µCi/mL [^3^H]-E3S (represented as E3S/[^3^H]-E3S). E3S was found to exhibit a biphasic pharmacokinetic profile with short distribution and long elimination half-lives (t_1/2α_ (h) = 0.04±0.01, t_1/2β_ (h) = 17.0±3.0). The AUC, CL and Vss were 222.8±49.4%I.D./mL*h, 0.5±0.1 mL/h and 10.6±1.5 mL, respectively.

### Biodistribution of ^3^H-E3S in Non-tumour and Tumour Bearing Mice

**Figure 2:**
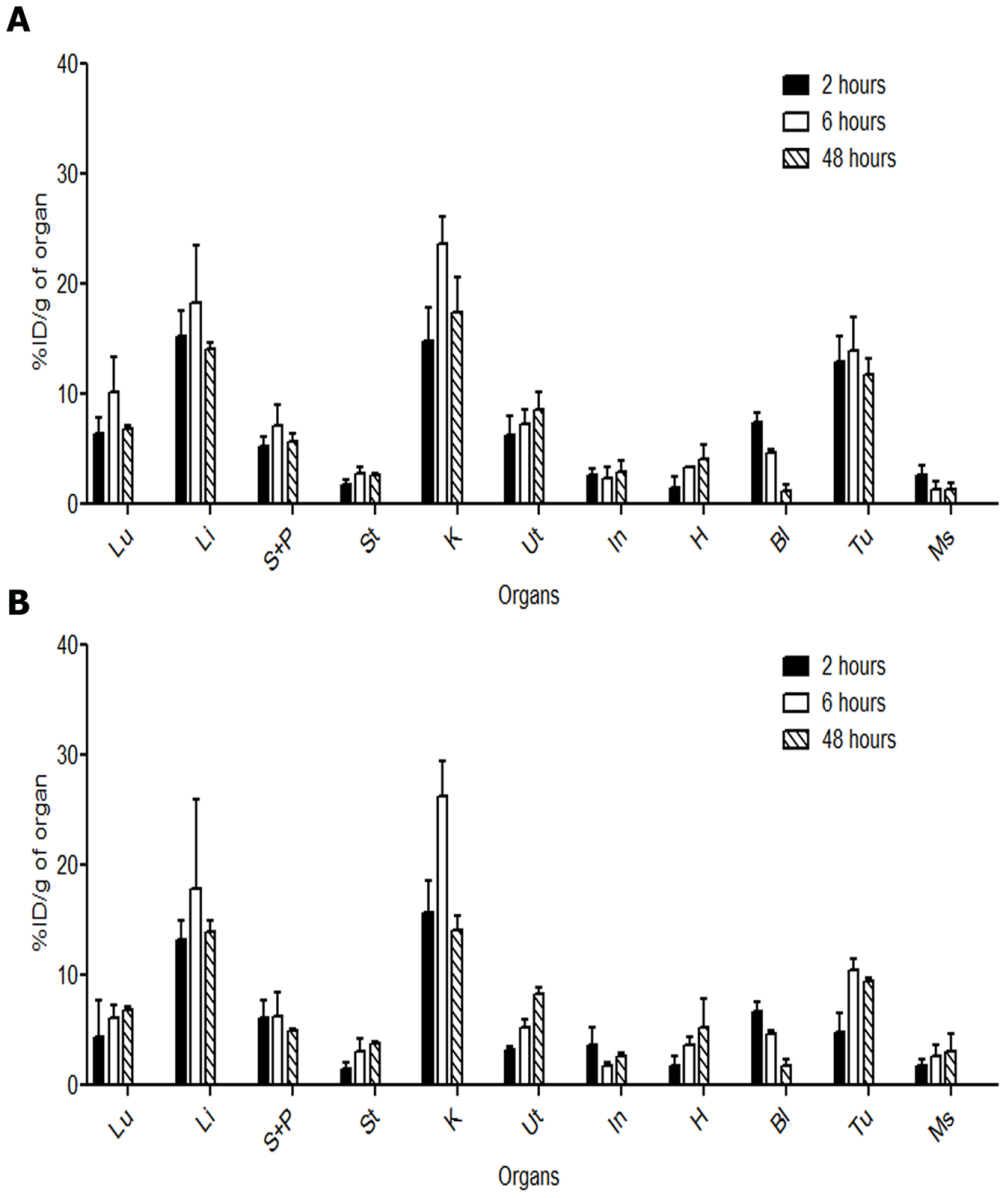
Biodistribution of E3S/[^3^H]-E3S. Biodistribution of E3S/[^3^H]-E3S at 2 h, 6 h and 48 h p.i. in mice bearing MCF-7 (A) and MDA-MB-231 (B) xenograft expressed as % injected dose per gram (%ID/g). (Lu: Lung, Li: Liver, S+P: Spleen and Pancreas, St: Stomach, K: Kidneys, Ut: Uterus, In: Intestine, H: Heart, Bl: Blood, T: Tumour, Ms: Muscle).

**Figure 3:**
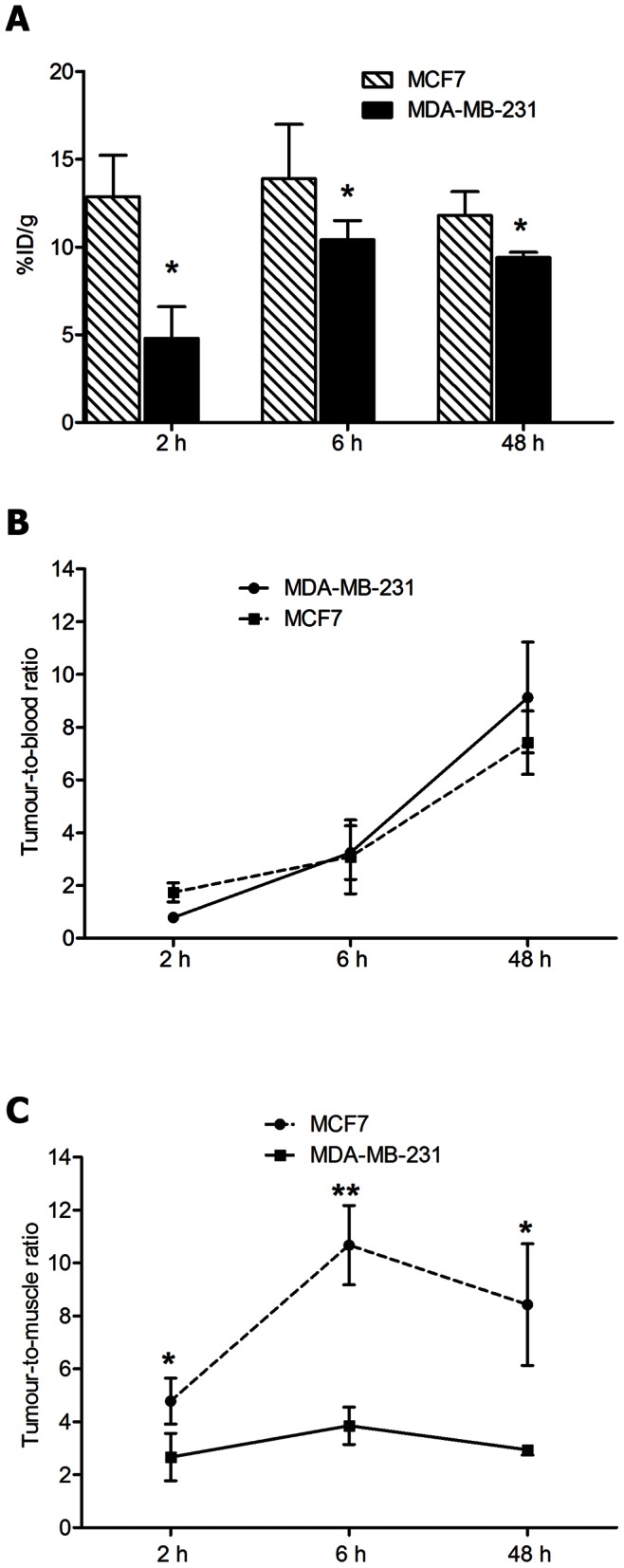
Tumour uptake (A), tumour-to-blood ratios (B), and tumour-to-surrounding muscle ratios (C) of E3S. Tumour uptake (A), tumour-to-blood ratios (B), and tumour-to-surrounding muscle ratios (C) of E3S in mice bearing MCF-7 (hormone dependent) and MDA-MB-231 (non-hormone dependent) xenografts expressed as % injected dose per gram (%ID/g) at 2 h, 6 h and 48 h p.i. Uptake of E3S/[^3^H]-E3S was significantly higher in hormone dependent MCF-7 xenografts at all time points. Statistically significant (*p*<0.05) difference is also observed in the ratio of the tumour uptake and the surrounding muscle uptake between the two xenograft models. *p<0.05, **p<0.01.

The biodistribution of ^3^H-E3S was evaluated in non-tumour bearing mice as well as in hormone dependent (MCF-7) and non-hormone dependent (MDA-MB-231) xenograft mouse models. The biodistribution of E3S in non-tumour bearing mice was similar to that observed in the tumor bearing mice (Figure S1). Fig. 2A and 2B show the normal tissue uptake of E3S in MCF-7 and MDA-MB-231 tumour bearing mice following an i.v. administration of 0.25 nmoles/kg of E3S/[^3^H]-E3S. Tissue accumulation in all organs was similar for mice bearing MCF-7 and MDA-MB-231 xenografts. The peak levels in liver and kidneys were observed at 6 h p.i. with 18.3±5.3%ID/g and 23.5±2.5%ID/g, respectively, in MCF-7 tumour bearing mice, and 17.8±8.1%ID/g and 26.2±3.2%ID/g, respectively, in MDA-MB-231 tumour bearing mice. Given that the uterus is a hormone dependent organ, significant uterine uptake was also observed in both xenograft models. The uterine uptake of E3S in MCF-7 tumour bearing mice was 6.2±1.8, 7.3±1.4 and 8.6±1.6% I.D./g at 2 h, 6 h and 48 h post injection, respectively. For MDA-MB-231 tumour bearing mice it was 3.2±0.3, 5.3±0.6 and 8.3±0.5%I.D./g at 2 h, 6 h and 48 h post injection, respectively. It is likely that mice bearing MCF-7 tumours had better developed uteri, given that they were supplemented with estradiol to support tumour growth. This could potentially explain the difference observed in the uterine E3S uptake between the two models.”Fig. 3A shows E3S tumour uptake in MCF-7 and MDA-MB-231 xenografts. In hormone dependent (MCF-7) xenografts the tumour uptake reached a high level at the early time point (2 h p.i.; 12.9±2.4% ID/g) and did not significantly change at 6 h (13.9±3.1% ID/g) (*p* = 0.67) or 48 h (11.8±1.4% ID/g) (*p* = 0.53) p.i. In contrast, the tumour uptake of E3S in the non-hormone dependent xenograft model (MDA-MB-231) increased from 4.8±1.8% ID/g at 2 h p.i. to 10.4±1.1% ID/g at 6 h p.i. (*p* = 0.01) and did not significantly change at 48 h (9.4±0.3% ID/g) (*p* = 0.20). There was a significant difference in tumour uptake between the two xenograft models at 2 h (*p* = 0.01), 6 h (*p* = 0.04) and 48 h p.i. (*p* = 0.02). The highest tumour-to-blood ratios were observed at 48 h p.i. as a result of clearance of E3S from the circulation (Fig. 3B). Fig. 3C shows the tumour to surrounding muscle tissue ratio for both xenografts. The tumour-to-muscle ratio was significantly higher in the MCF-7 xenograft model in comparison to the MDA-MB-231 model at all time points. The muscle uptake at 2 h, 6 h and 48 h p.i. was 2.6±0.4, 1.3±0.7 and 1.4±0.5%ID/g, respectively, in the MCF-7 tumour bearing mice and 1.8±0.6, 2.7±0.9 and 3.1±1.6%ID/g, respectively, in the MDA-MB-231 tumour bearing mice. There were no significant differences [p = 0.33(2 h); p = 0.29(6 h); p = 0.37(48 h)] in the muscle uptake between the two xenograft models.

**Figure 4:**
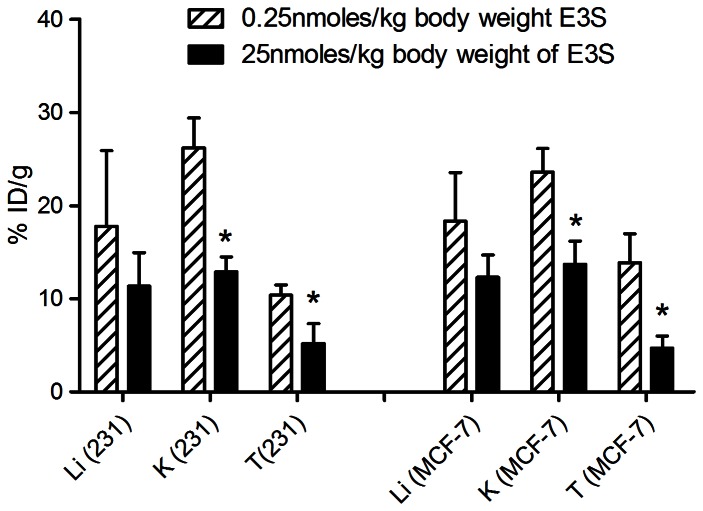
Tumour and tissue distribution of E3S/[^3^H]-E3S, following a blocking dose, in mice bearing xenografts. Statistically significant (*p*<0.05) differences in uptake were observed in the tumour and kidney in both MCF-7 and MDA-MB-231 xenograft models *p<0.05. (Li (231): Liver from MDA-MB-231 tumour bearing mice, K (231): Kidneys from MDA-MB-231 tumour bearing mice, T (231): MDA-MB-231 xenograft, Li (MCF-7): Liver from MCF-7 tumour bearing mice, K (MCF-7): Kidney from MCF-7 tumour bearing mice, T (MCF-7): MCF-7 xenograft).

To determine if the high uptake observed in the tumour, kidneys and liver was mediated through an active carrier process, a blocking study was performed in which MCF-7 and MDA-MB-231 tumour bearing mice were injected via the tail vein with 100-fold excess E3S (25 nmole/kg) 2 h prior to administration of 0.25 nmole/kg E3S/[^3^H]-E3S. Fig. 4 shows the tissue (liver, kidney and tumour) uptake of E3S/[^3^H]-E3S in both xenograft models. When mice were pre-dosed with 100-fold excess E3S, there was a 3-fold (*p* = 0.01) and 2-fold (*p* = 0.02) reduction in tumour uptake in MCF-7 and MDA-MB-231 xenografts, respectively. Similarly, there was a 1.7-fold (p = 0.01) and 2.0-fold (p = 0.003) reduction in kidney uptake in MCF-7 and MDA-MB-231 tumour bearing mice, respectively. A slight but insignificant decrease in liver uptake was also observed in both xenograft models.

### *Ex vivo* Tumour Cell Uptake of E3S in MCF-7 and MDA-MB-231 Xenografts

**Figure 5:**
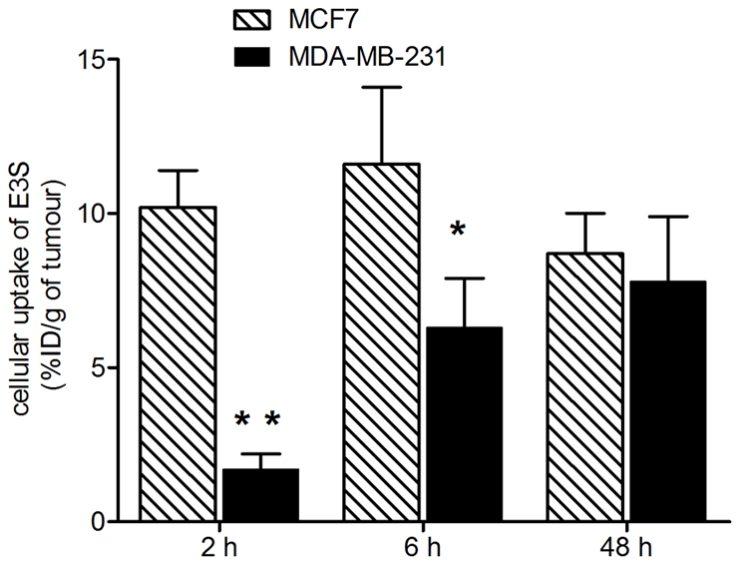
*Ex vivo* cellular uptake of E3S in MCF-7 and MDA-MB-231 xenografts. Significant differences in cellular uptake of ^3^H-E3S between the MCF-7 and MDA-MB-231 xenografts were observed at 2 h (***p<0.001) and 6 h (*p<0.05) p.i. but not at 48 h p.i.

The *ex vivo* cellular uptake of E3S was evaluated in the MCF-7 and MDA-MB-231 tumours harvested at 2h, 6h and 48h p.i. As shown in Fig. 5, cellular uptake of E3S was significantly higher in the MCF-7 tumours than in the MDA-MB-231 tumours at 2 h (6-fold higher; *p* = 0.0003) and 6 h (1.8-fold higher; *p* = 0.04) p.i. However, there was no significant difference in the cellular levels at 48 h p.i. suggesting saturable uptake in both xenografts at this time point.

### Metabolite Analysis of Plasma for E3S, Estrone and Estradiol

For the biodistribution studies the levels of E3S were assessed in tumours and tissues by scintillation counting with the assumption that the ^3^H-E3S had not been metabolized to ^3^H-estrone or ^3^H-estradiol. Metabolite analysis of plasma collected at 2, 6 and 48 h was conducted by HPLC. Fig. S2 shows a representative HPLC chromatogram of the metabolism profile of E3S in plasma at 48 h p.i. Overall there was an increase in the metabolism of E3S to estrone and estradiol with time with the highest concentrations of estrone and estradiol being observed at 48 h p.i. (Table S1).

### Determination of OATP1A2 Expression and Blood Microvessel Density in MCF-7 and MDA-MB-231 Xenografts by Immunohistochemistry

**Figure 6:**
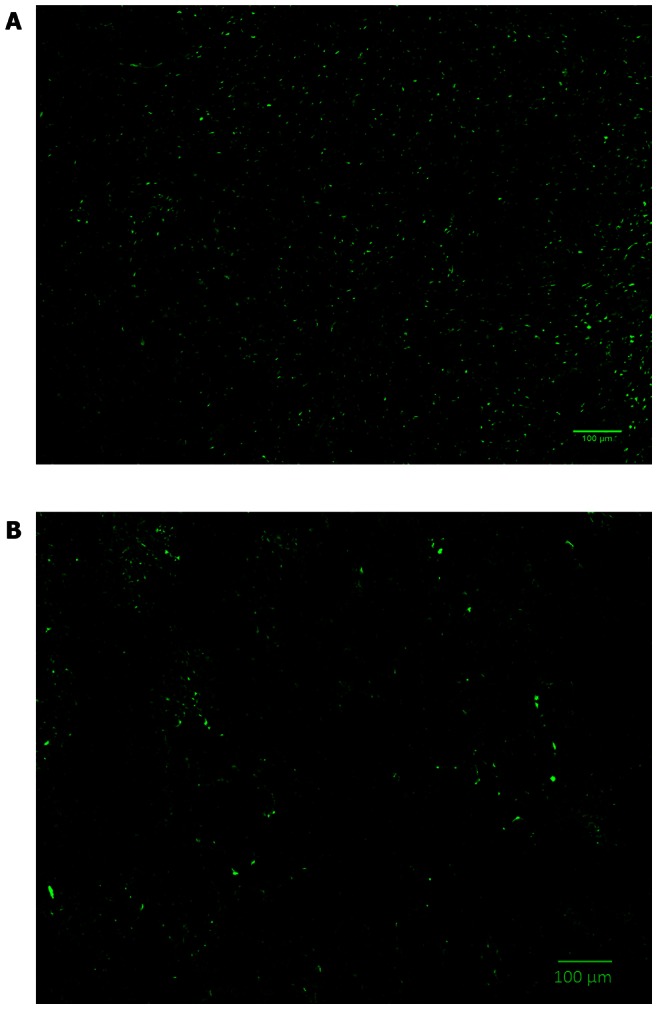
OATP1A2 expression in MCF-7 (A) and MDA-MB-231 (B) xenograft sections. A statistically significant (p<0.05) difference was observed in the OATP1A2 expression between the MCF-7 and MDA-MB-231 tumour xenografts expressed in % area (**p<0.005).

Fig. 6A and 6B shows representative sections of the MCF-7 and MDA-MB-231 xenografts that were stained for OATP1A2. Significantly higher OATP1A2 expression (p = 0.002) was observed in the sections from the MCF-7 xenografts (4.59±0.63% area) as compared to the sections from the MDA-MB-231 xenografts (2.78±0.87% area). OATP1A2 protein expression was also confirmed in tumour tissues from MCF-7 and MDA-MB-231 tumours and significantly (p = 0.002) higher OATP1A2 protein expression was observed in the MCF-7 tumour tissues (Fig. S3). There was no significant difference (p = 0.37) in the microvessel density between the MCF-7 (4.6±0.3% area) and MDA-MB-231 (4.86±0.58% area) tumour xenografts. Fig. S4A and S4B show representative sections of the MCF-7 and MDA-MB-231 xenografts that were stained for CD31. Fig. S5A, B represents positive and negative controls for OATP1A2 and CD31 stained blood vessels.

## Discussion

The current studies focus on assessing the potential of E3S as a novel ligand for targeting OATPs expressed in breast tumour tissue. The following OATP isoforms OATP1A2, OATP1B1, OATP1B3, OATP1C1, OATP2B1, OATP3A1, OATP4A1 and OATP4C1 have been reported to recognize E3S as a substrate [25]. Although there have been previous studies reporting over expression of some of these OATPs in breast tumour tissue compared to normal tissue [17], [18], [26], [27], the potential of OATP as a novel molecular target has not been investigated *in vivo*.

As E3S is known to be the predominant source of estrogen in hormone dependent breast tumours detected in post-menopausal women [28], ovariectomized mice with minimal levels of circulating plasma estradiol [29] were employed for these studies. The pharmacokinetic profile of E3S includes a considerably long plasma half-life (t_1/2β_) of 17.0±3.0 h that can be explained by the proposed futile cycling of E3S [30], [31]. Back et al. had reported t_1/2β_ of E3S to be 6 h in rats and they indicated entero-hepatic circulation to be an important factor in determining the pharmacokinetics of E3S [32]. The difference in the t_1/2β_ values obtained in that study and the current study may be attributed to the different species of animals employed.

Tumour accumulation of E3S in hormone dependent MCF-7 xenografts was significantly higher than that in hormone independent MDA-MB-231 xenografts. Specifically, tumour uptake of E3S at early time points (i.e. 2 h p.i) was 2.7 fold higher in the MCF-7 tumours, while at later time points (i.e. 6 h and 48 h p.i.) it was 1.3 fold higher. Our previous evaluation of the transport kinetics of E3S *in vitro* indicated that the affinity of cells for E3S is higher in the MCF-7 cells (*K_m_* = 6.5±1.8 µM) compared to the MDA-MB-231 cells (*K_m_* = 46.9±1.7 µM) [16], [33]. These *in vitro* observations suggest that the higher affinity of MCF-7 cells for E3S could be the reason for the 2.7 fold higher E3S uptake observed in MCF-7 xenografts at 2 h p.i. Furthermore, in agreement with previous *in vitro* studies that reported similar E3S transport capacity in both MCF-7 (*V_max_ = *66±8.5 pmol/mg protein/min) and MDA-MB-231 (*V_max_ = *27.3±6.2 pmol/mg protein/min) cells [16], it was observed that the levels of tumour uptake achieved at the later time point (i.e. 48 h p.i.) were similar in both xenograft models with slightly higher uptake in the MCF-7 xenografts. Analogous to total tumour uptake, *ex vivo* tumour cell uptake at 2 h p.i. was 6 fold higher in MCF-7 tumour cells in comparison to MDA-MB-231 tumour cells. Once again this may be attributed to the higher affinity of MCF-7 cells for E3S. Moreover, given that the E3S transport capacity is similar in both cell lines, at 48 h p.i., there was no significant difference in E3S uptake in the MCF-7 and MDA-MB-231 tumour cells. Thus the *in vivo* tumour uptake and *ex vivo* tumour cell uptake of E3S in MCF-7 and MDA-MB-231 xenografts are in good agreement with previous reports on the *in vitro* transport kinetics for E3S in MCF-7 and MDA-MB-231 cells. In addition, the blocking studies revealed significantly lower tumour uptake of E3S in both xenograft models post administration of excess (100 fold) E3S, suggesting, that tumour uptake of E3S was primarily an active carrier mediated process. “Pasqualini et al. have previously reported increased sulphatase activity in intact MCF-7 cells relative to the level of activity in MDA-MB-231 cells. This increased sulphatase activity resulted in increased conversion of E3S to estradiol [34]. This may create intra-cellular “sink conditions”, which could in turn lead to higher transporter mediated E3S uptake in the MCF-7 xenografts in comparison to the MDA-MB-231 xenografts. Future studies will examine E3S uptake in MCF-7 and MDA-MB-231 xenografts in the presence of a physiological inhibitor of estrone sulphatase in order to elucidate the contribution of the sulphatase enzyme in E3S tumour uptake.

Previously reported mRNA and protein expression of OATP1A2, OATP1B1, OATP1B3, OATP2B1, OATP3A1 and OATP4A1 in MCF-7 and MDA-MB-231 cells suggest that OATP1A2 is one of the most significant isoforms expressed in both cell lines [16], [18]. Hence, OATP1A2 expression was examined in tumour sections from both xenografts to determine if this isoform contributed towards the active carrier mediated uptake of E3S in tumours. The significantly higher level of OATP1A2 expression observed in the MCF-7 xenograft sections indicates that the higher uptake of E3S observed in these tumours may in part be due to the over-expression of this isoform. It is possible that the different OATP isoforms that transport E3S, exhibit a different binding affinities for E3S. Further investigation is necessary in order to compare the expression and binding affinities of other OATP isoforms among these two xenograft models. It is postulated that the higher E3S uptake observed in the MCF-7 tumours is due to one or a combination of the following factors: higher affinity of MCF-7 cells for E3S, higher levels of OATP1A2 expression in MCF-7 xenografts and an increased sulphatase activity in MCF-7 cells.

Microvessel densities were also compared between both xenografts to evaluate the role of vascularization in tumour uptake of E3S and no significant difference (p = 0.37) was observed in CD31 staining between the MCF-7 and MDA-MB-231 xenografts. This suggests that the difference in tumour uptake is not contributed by difference in tumour vascularisation.

The high tumour to blood and tumour to muscle ratios suggest that E3S may have promise as an imaging agent for detection of breast cancer. Although there was no significant difference in the tumour to blood ratios among the two xenograft models, the tumour to muscle ratios were significantly higher in the MCF-7 xenografts, suggesting that E3S would be a better targeting ligand for hormone dependent breast tumours. However, the use of E3S to target diagnostic or therapeutic agents to breast cancer could also result in toxicity given the uptake of E3S in normal tissues such as the liver and kidneys. Furthermore, the blocking studies demonstrated that the liver and kidney uptake was also an active carrier mediated process as E3S kidney uptake was significantly reduced following administration of excess E3S. Although liver uptake could not be significantly reduced following the blocking dose, there was 3.5 and 2.2 fold reduction in liver uptake in MCF-7 and MDA-MB-231 tumour bearing mice, respectively. Many oatp (representation for rodent OATP) isoforms have been reported to be expressed in liver and kidneys where they facilitate the clearance of various endogenous molecules and xenobiotics [35]–[38]. The specific carrier mediated E3S uptake observed in the liver and kidneys could be mediated by oatp as well.

Overall, the promising tumour uptake, tumour-to-blood and tumour-to-muscle ratios observed for E3S in MCF-7 tumour bearing mice, indicate that this substrate may be a promising ligand for targeting hormone dependent breast cancer. In particular, the relatively high tumour-to-blood ratio suggests the potential of this molecule for imaging applications such as cancer detection and diagnosis. Currently imaging agents like ^18^F-fluoroestradiol (^18^F-FES) and ^123^I-estradiol are used for PET and SPECT imaging [39]–[42], respectively, of hormone dependent cancers. However, the effective use of these agents is limited due to loss or mutation of estrogen receptor (ER) expression [43], [44]. The approach of targeting OATPs in hormone dependent breast tumours, with E3S as the targeting ligand, could potentially address the limitations of ER mediated imaging [45], [46].

## Supporting Information

Figure S1 **Biodistribution of E3S/[^3^H]-E3S.** Biodistribution of E3S/[^3^H]-E3S at 2 h, 6 h and 48 h p.i. in non-tumour bearing mice expressed as % injected dose per gram (%ID/g). (Lu: Lung, Li: Liver, S+P: Spleen and Pancreas, St: Stomach, K: Kidneys, Ut: Uterus, In: Intestine, H: Heart, Bl: Blood)
(TIF)

Figure S2 **HPLC analyses of estrone-3-sulphate (E3S) and its metabolites, estrone and estradiol, in plasma at 48 h p.i.** UV channel represents reversed-phase HPLC (see methods section for HPLC conditions) chromatograms of E3S, estrone and estradiol. For metabolite analyses plasma samples of mice injected with E3S/[^3^H]-E3S, were collected at different time points (2, 6 or 48 h) and were spiked with 0.625 mg/mL of E3S, estrone and estradiol, prior to solid phase extraction. HPLC eluates were collected every min for 50 min and the radioactivity in the samples were counted and plotted. Peak area of the radiometric channel represents the contribution of the E3S and its metabolites in plasma.
(TIF)

Figure S3 **Immunoblot and densitometric analysis of OATP1A2 transporters.** Protein expression of OATP1A2 was investigated in xenograft tissues **(**MDA-MB-231: represented by lanes **2,3,4** and MCF7: represented by lanes **5,6,7**). To determine the specificity of the respective antibody used, a positive control cell line over expressing the OATP1A2 transporter (HEK293/OATP1A2: represented by lane **1**) was included in the blot. Results of the densitometric analysis were expressed as mean ± SD of three separate tumours for each xenograft model. A significant difference in protein expression was observed between the two xenograft tissues. **p = 0.002 is considered to be statistically significant.
(TIF)

Figure S4 **Functional and non-functional vessels (stained with CD31) in MCF-7 (A) and MDA-MB-231 (B) xenograft sections.** No statistically significant difference was observed in the microvessel density between the MCF-7 and MDA-MB-231 tumour xenografts.
(TIF)

Figure S5 **Positive and negative controls for OATP1A2 (A) and CD31 (B).** Human brain tissue and bladder tumour tissue sections were used as positive controls to determine specificity of antibodies used for OATP1A2 and CD31 staining, respectively. The same tissues were stained with secondary antibody (without any primary antibody) to determine non-specific binding and these served as negative controls.
(TIF)

Table S1 **Plasma analysis of estrone-3-sulphate (E3S) and its metabolites estrone and estradiol in methanol, hexane and water fractions collected, following solid phase extraction (SPE).** The plasma samples were collected at 2 h, 6 h and 48 h post-injection (p.i.) from MCF-7 and MDA-MB-231 tumour bearing mice and were then subjected to SPE.
(DOCX)
