# Supplementary figures and images for: Estrone-3-Sulphate, a Potential Novel Ligand for Targeting Breast Cancers
Source: PLoS One. 2013 May 22;8(5):e64069. doi: 10.1371/journal.pone.0064069 (PMC3661587; doi:10.1371/journal.pone.0064069)

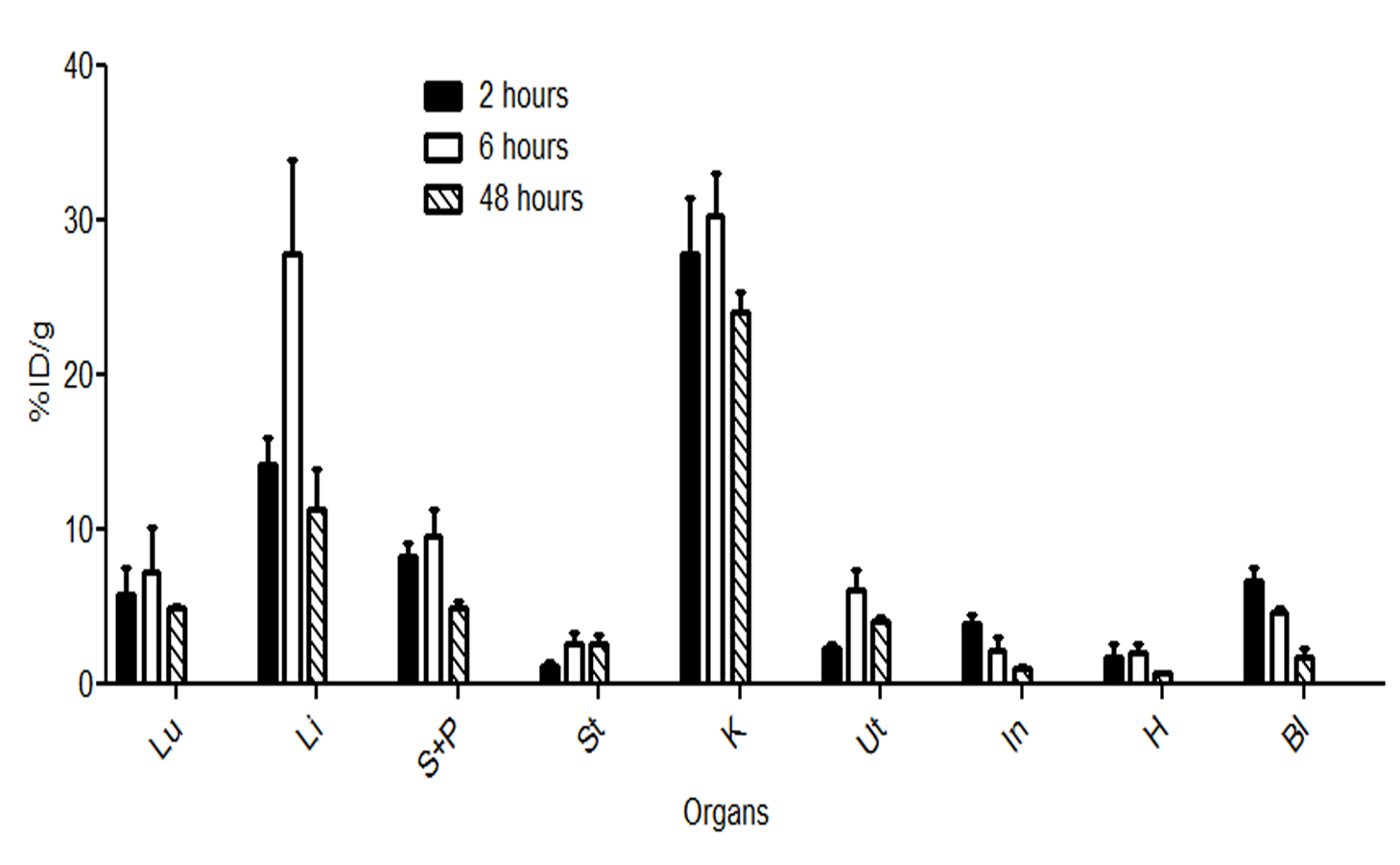

Supplement: Figure S1 — Biodistribution of E3S/[3H]-E3S. Biodistribution of E3S/[3H]-E3S at 2 h, 6 h and 48 h p.i. in non-tumour bearing mice expressed as % injected dose per gram (%ID/g). (Lu: Lung, Li: Liver, S+P: Spleen and Pancreas, St: Stomach, K: Kidneys, Ut: Uterus, In: Intestine, H: Heart, Bl: Blood) (TIF) [file pone.0064069.s001.tif]

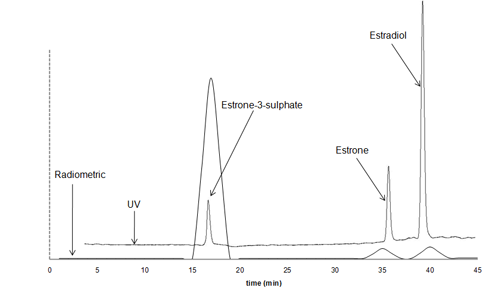

Supplement: Figure S2 — HPLC analyses of estrone-3-sulphate (E3S) and its metabolites, estrone and estradiol, in plasma at 48 h p.i. UV channel represents reversed-phase HPLC (see methods section for HPLC conditions) chromatograms of E3S, estrone and estradiol. For metabolite analyses plasma samples of mice injected with E3S/[3H]-E3S, were collected at different time points (2, 6 or 48 h) and were spiked with 0.625 mg/mL of E3S, estrone and estradiol, prior to solid phase extraction. HPLC eluates were collected every min for 50 min and the radioactivity in the samples were counted and plotted. Peak area of the radiometric channel represents the contribution of the E3S and its metabolites in plasma. (TIF) [file pone.0064069.s002.tif]

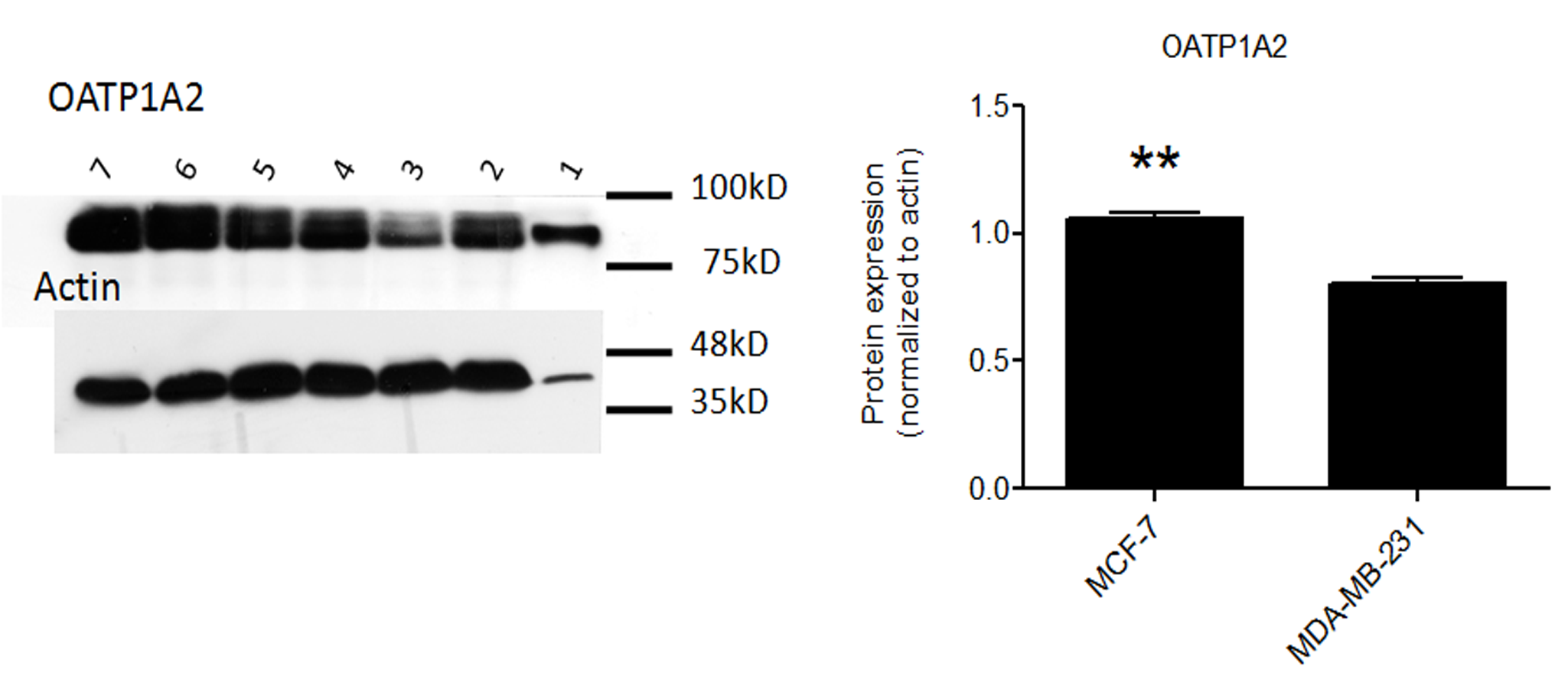

Supplement: Figure S3 — Immunoblot and densitometric analysis of OATP1A2 transporters. Protein expression of OATP1A2 was investigated in xenograft tissues (MDA-MB-231: represented by lanes 2,3,4 and MCF7: represented by lanes 5,6,7). To determine the specificity of the respective antibody used, a positive control cell line over expressing the OATP1A2 transporter (HEK293/OATP1A2: represented by lane 1) was included in the blot. Results of the densitometric analysis were expressed as mean ± SD of three separate tumours for each xenograft model. A significant difference in protein expression was observed between the two xenograft tissues. **p = 0.002 is considered to be statistically significant. (TIF) [file pone.0064069.s003.tif]

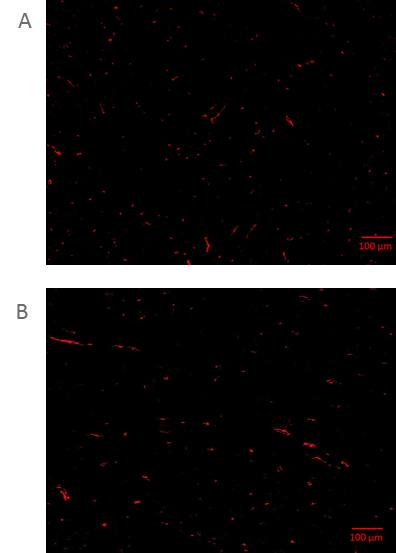

Supplement: Figure S4 — Functional and non-functional vessels (stained with CD31) in MCF-7 (A) and MDA-MB-231 (B) xenograft sections. No statistically significant difference was observed in the microvessel density between the MCF-7 and MDA-MB-231 tumour xenografts. (TIF) [file pone.0064069.s004.tif]

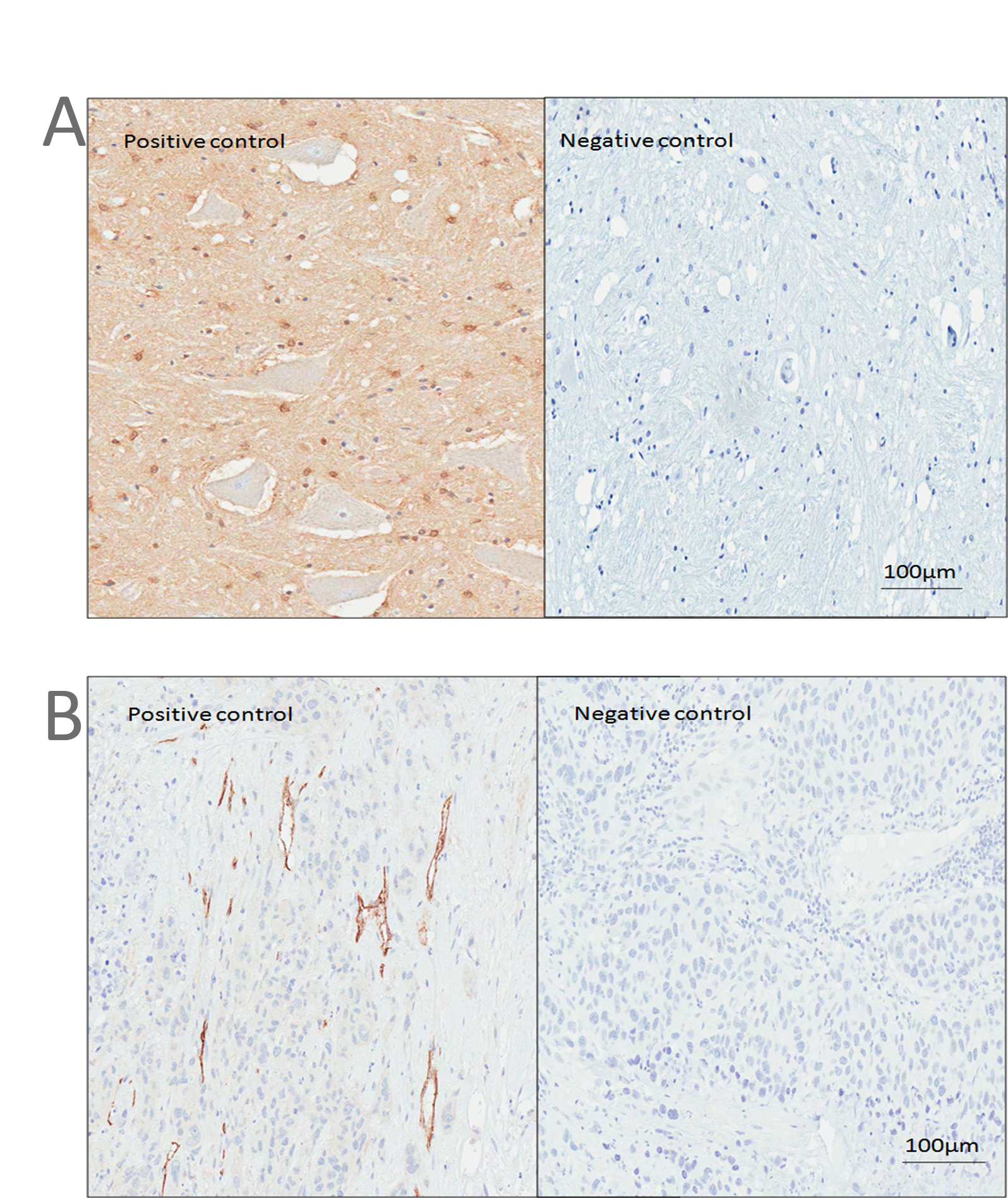

Supplement: Figure S5 — Positive and negative controls for OATP1A2 (A) and CD31 (B). Human brain tissue and bladder tumour tissue sections were used as positive controls to determine specificity of antibodies used for OATP1A2 and CD31 staining, respectively. The same tissues were stained with secondary antibody (without any primary antibody) to determine non-specific binding and these served as negative controls. (TIF) [file pone.0064069.s005.tif]
