# Supplementary material for: Estrone-3-Sulphate, a Potential Novel Ligand for Targeting Breast Cancers
Source: PLoS One. 2013 May 22;8(5):e64069. doi: 10.1371/journal.pone.0064069 (PMC3661587; doi:10.1371/journal.pone.0064069)
Supplement: Table S1 — Plasma analysis of estrone-3-sulphate (E3S) and its metabolites estrone and estradiol in methanol, hexane and water fractions collected, following solid phase extraction (SPE). The plasma samples were collected at 2 h, 6 h and 48 h post-injection (p.i.) from MCF-7 and MDA-MB-231 tumour bearing mice and were then subjected to SPE. (DOCX) [file pone.0064069.s006.docx]

SUPPLEMENTARY TABLE

Table S1: Plasma analysis of estrone-3-sulphate and its metabolites, estrone and estradiol.

| Plasma  (n=3 at each time point) | Estrone-3-sulphate  (% of total radioactivity) | Estrone  (% of total radioactivity) | Estradiol  (% of total radioactivity) |
| --- | --- | --- | --- |
| 2h-MeOH extract | 90±2.7% | 1.02±0.24% | 2.2±1.23% |
| 2h- Hexane extract | 1.2±0.5% | n.d. | n.d. |
| 2h-Water extract | 2.1±1.2% | n.d. | n.d. |
| 6h-MeOH extract | 92±1.2% | 1.7±0.8% | 3.2±0.2% |
| 6h- Hexane extract | 0.87±0.24% | n.d. | n.d. |
| 6h-Water extract | n.d. | n.d. | n.d |
| 48h-MeOH extract | 84.3±5.4% | 1.4±0.62% | 3.2±0.45% |
| 48h- Hexane extract | 1.56±0.8% | 1.2±0.8% | n.d |
| 48h-Water extract | n.d. | n.d. | n.d. |
